# Innate Lymphoid Cells in HIV/SIV Infections

**DOI:** 10.3389/fimmu.2017.01818

**Published:** 2017-12-13

**Authors:** Spandan V. Shah, Cordelia Manickam, Daniel R. Ram, R. Keith Reeves

**Affiliations:** ^1^Center for Virology and Vaccine Research, Beth Israel Deaconess Medical Center, Harvard Medical School, Boston, MA, United States

**Keywords:** innate lymphoid cells, innate immunity, HIV infections, SIV, mucosal immunity

## Abstract

Over the past several years, new populations of innate lymphocytes have been described in mice and primates that are critical for mucosal homeostasis, microbial regulation, and immune defense. Generally conserved from mice to humans, innate lymphoid cells (ILC) have been divided primarily into three subpopulations based on phenotypic and functional repertoires: ILC1 bear similarities to natural killer cells; ILC2 have overlapping functions with TH2 cells; and ILC3 that share many functions with TH17/TH22 cells. ILC are specifically enriched at mucosal surfaces and are possibly one of the earliest responders during viral infections besides being involved in the homeostasis of gut-associated lymphoid tissue and maintenance of gut epithelial barrier integrity. Burgeoning evidence also suggests that there is an early and sustained abrogation of ILC function and numbers during HIV and pathogenic SIV infections, most notably ILC3 in the gastrointestinal tract, which leads to disruption of the mucosal barrier and dysregulation of the local immune system. A better understanding of the direct or indirect mechanisms of loss and dysfunction will be critical to immunotherapeutics aimed at restoring these cells. Herein, we review the current literature on ILC with a particular emphasis on ILC3 and their role(s) in mucosal immunology and the significance of disrupting the ILC niche during HIV and SIV infections.

## Introduction

Innate lymphoid cells (ILC) encompass a broad diversity of cell types including the nominal subtypes ILC1, ILC2, ILC3, and in some descriptions also include traditional natural killer (NK) cells and lymphoid tissue inducer cells, all of which arise from a common lymphoid progenitor. A common consensus in the field favors grouping of these cells based on the dependence on transcription factors, as well as by production of major cytokine classes (1–4). ILC1 and NK cells rely on the transcription factor T-bet and produce type I cytokines, such as IFN-γ and TNF-α, but notably ILC1 lack the complex cytotoxic functions inherent to NK cells. ILC2 are classified by their dependence on GATA3, and their production of IL-5 and IL-13 (5, 6), and finally ILC3 are generally identified by their dependence on RORγt and AHR, and secretion of IL-17 and IL-22 (7). Interestingly, through the expression of their respective cytokines and dependence on transcription factors for their development, the three ILC groups (1–3) show strong commonalities with TH1, TH2, and TH17/TH22 cells, respectively (8). It is important to note that the classification scheme remains somewhat fluid and grouping is not absolute, as NK cells and ILC1 do not always require T-bet (9), and ILC2 and ILC3 can both convert to ILC1 (10), underscoring the inherent plasticity of these cell types. To further complicate matters, innate subsets of lymphoid cells may also include mucosal-associated invariant T cells (11), which express a semi-invariant T cell receptor and defined phenotypically as CD3^+^Vα7.2 TCR^+^CD161^high^ cells in humans (12, 13). In addition to their cellular and functional plasticity, ILC have a wide tissue distribution and thus are thought to be some of the earliest responders to infections and other inflammatory stimuli, but the full mechanisms involved are still poorly understood. Striking observations have revealed that lentiviral infection leads to the depletion of functional ILC3 in gut mucosae (14–16), and increased microbial translocation from the gut lumen and an overt disruption of epithelial tissue integrity in HIV^+^ individuals is linked to a massive loss of IL-17-producing gut-resident lymphocytes (17). It is now becoming increasingly clear that reduced IL-17 and IL-22 production during infection cannot be attributed solely to the loss of TH17/TH22 cells and that early depletion of ILC may also contribute to this process.

## ILC Phenotypes and Distribution

Although ILC are typified by their unique plasticity and their descriptive definitions are somewhat fluid, some generally accepted phenotypic nomenclatures have been established. ILC are usually identified as negative for common lymphocyte lineage markers (Lin^−^) that are otherwise distinct from NK cells and can usually be distinguished as such by higher expression of the IL-7 receptor, CD127 (1–4). However, even these definitions can vary significantly as “Lin” markers differ depending on the animal species (Table 1). For instance, in mice the Lin group may include CD3, CD4, CD8, CD11b, CD11c, CD14, CD19, B220, FcεRI, TER119 antigen, and GR1, whereas in humans, the Lin group may include CD1a, CD3, CD11c, CD14, CD16, CD19, CD34, CD123, TCRαβ, TCRγδ, BDCA2, and FcεRI. Burkhard et al. (18) recommends using CD5 marker in order to exclude small levels of contaminating T cells, especially for analyzing ILC3 populations. Regardless, these exclusion criteria remove T, B, NK, and dendritic cells, as well as other myeloid/granulocyte-derived cells and stem cells. ILC in rhesus macaque models align most closely to patterns seen in humans, but partly due to variability in cross-reactive reagents, may be more simply defined by excluding CD3, FcεRI, CD14, CD20, and NK cell-related markers, such as NKG2A or NKp46 (15, 16, 19). It is also important to note that exclusion of Lin markers may vary significantly between laboratories. Several other factors are used to characterize ILC, including the presence of various cytokines mentioned above and utilization of key transcription factors and receptors (1–5, 20). The co-expression of NKp46 and NK1.1 classifies mouse ILC1 subsets including related NK cells from other ILC groups whereas the expression of transcription factors, namely T-bet and Eomes, can be used to distinguish ILC1 and NK cells from each other (21–23). Loosely, in mice, NK cells are T-bet^+^Eomes^+^ while ILC1 are T-bet^+^Eomes^−^ cells, although exceptions to this classification occur (9). Based on the nomenclature proposed by different reports (1, 2), ILC1 can be more comprehensively phenotyped as Lin^−^CD127^+^RORγt^−^T-bet^+^IL-1R^+^ cells in mice, and Lin^−^CD127^+^ICOS^+^RORγt^−^T-bet^+^IL-1R^+^ cells in humans. ILC2 are described as Lin^−^CD25^+^CD127^+^ICOS^+^THY1^+^SCA1^+^ST2^+^ cells in mice and Lin^−^CD25^lo^CD127^+^CD161^+^ICOS^+^CRTH2^+^ST2^+^ in humans. Similarly, ILC3 may be identified as Lin^−^CD25^+^CD127^+^CD117^+^THY1^+^NKp46^+/−^RORγt^+^IL-1R^+^ in mice and Lin^−^CD127^+^CD161^+/−^CD117^+^NKp46^+/−^NKp44^+/−^RORγt^+^IL-1R^+^IL-23R^+^ in humans. ILC may also be partially identified by receptors of cytokines to which they are responsive—IL-12Rβ2^+^ (ILC1), IL-17RB^+^ (ILC2), and IL-23R^+^ (ILC3), but due to issues with antibody specificity may best be shown molecularly or in functional assays. Collectively, these phenotypic descriptions of ILC populations continue to evolve, and while there is generally a good consensus about the definition of ILC2 and ILC3, what truly defines ILC1 is still somewhat unclear. Currently there are no unique markers or complete phenotypes that uniquely identify ILC1, and the field is still limited to their identification *via* exclusion criteria—i.e., cells that are not NK cells, ILC2, or ILC3. Functionally ILC1 are identified as IFN-γ-producing cells that are distinct from NK cells through their low cytotoxic potential. Understandably, these factors make the study of ILC1 particularly cumbersome. Indeed, a recent profiling of ILC across tissues using mass spectrometry by Simoni et al. (24) indicated lack of ILC1 as described previously by other groups (25, 26). Instead, they described a unique intra-epithelial ILC1-like cells (ieILC1) that matched the description by Fuchs et al. (27).

**Table 1:** Phenotypic markers and tissue distribution for innate lymphoid cell (ILC) groups.

	ILC1	ILC2	ILC3	Reference
Mouse^a^	Lin^−^CD127^+^RORγt^−^T-bet^+^IL-1R^+^IL-12Rb2^+^	Lin^−^CD25^+^CD127^+^ICOS^+^THY1^+^SCA1^+^ST2^+^IL-17Rb^+^	Lin^−^CD25^+^CD127^+^CD117^+^THY1^+^NKp46^+/−^RORγt^+^IL-1R^+^IL-23R^+^	(13, 20, 28–31)
Human^b^	Lin^−^CD127^+^ICOS^+^RORγt^−^T-bet^+^IL-1R^+^IL-12Rb2^+^	Lin^−^CD25^lo^CD127^+^CD161^+^ICOS^+^CRTH2^+^ ST2^+^IL-17Rb^+^	Lin^−^CD127^+^CD161^+/−^CD117^+^NKp46^+/−^NKp44^+/−^RORγt^+^IL-1R^+^IL-23R^+^	(13, 20, 32–36)
Tissues distribution	Lungs, small intestines, blood, bone marrow, liver	Lungs, blood, bone marrow, skin, small intestines	Colon, small intestines, oral mucosae, lymph node, bone marrow, skin, spleen, thymus	(20, 25, 30, 32, 33, 35–39)

*^a^Lineage markers for mouse are CD3, CD4, CD8, CD11b, CD11c, CD14, CD19, B220, FcεRI, TER119 antigen, and GR1*.

*^b^Lineage markers for humans are CD1a, CD3, CD11c, CD14, CD16, CD19, CD34, CD123, TCRαβ, TCRγδ, BDCA2, and FcεRI*.

Although ILC are generally found systemically, they are disparately distributed by subpopulation and are particularly enriched in mucosal sites and secondary lymphoid organs (Table 1). ILC have been identified in the lungs (ILC1, ILC2), colon (ILC3), small intestine (ILC1, ILC3), oral mucosae (ILC3), as well as in bone marrow, blood (ILC1, ILC2), lymph nodes (ILC3), liver (ILC1), and even in embryonic tissues (40), although the ILC-related NK cells tend to be much more broadly distributed (8). How ILC populations are maintained and replenished is unfortunately not well defined. Tissue-resident ILC predominantly replenish by self-renewal (40), though evidence suggests that common precursor cells from the bone marrow, or elsewhere, may also contribute to ILC homeostasis *via* cell recruitment (22, 41).

## Role of ILC in Gastrointestinal (GI)-Related Diseases and Repair

Innate lymphoid cell populations are constitutively present in the GI tract and lymphoid tissues but differ in their compartmental distribution (42, 43). In healthy humans, ILC1 are the major population in the upper compartment of the GI tract while ILC3 are elevated in ileum and colon (44). The local distribution of ILC within the GI tract also differ—ILC1 predominate the intra-epithelial compartment of the intestine (27, 41, 42), while ILC2 are present in fat-associated lymphoid clusters in the intestinal mesentery and in significant numbers in lamina propria of small intestine where ILC3 is the dominant population (44–46). ILC3 are also enriched in the isolated lymphoid follicles, crypto- patches, and perifollicular area of Peyer’s patches at steady state (20, 47).

In the healthy gut, ILC3 are thought to be one of the major cell populations contributing to overall homeostasis. This is, in part, because ILC3 produce large quantities of IL-22 and IL-17 (48), and directly interact with intestinal epithelial cells to maintain an intact barrier and modulate inflammation (49, 50). IL-22 protects intestinal epithelium from inflammation and promotes wound healing by inducing STAT-3 dependent increases in production of antimicrobials by epithelial cells and epithelial cell proliferation, thus maintaining barrier integrity (51–53). In a mouse model of dextran sulfate sodium-induced ulcerative colitis, microinjection-based gene delivery of IL-22 ameliorated local inflammation through activation of STAT-3 in colonic epithelial cells, stimulation of mucus production, and goblet cell restitution (54). In IL-22^−/−^ mice, increased intestinal damage, bacterial burden, and mortality was observed on infection with *Citrobacter rodentium* (52), and in humans, IL-22 has been shown to protect intestinal epithelium in IBD (55). Specifically, IL-23 responsive, IL-17/22-producing ILC protected intestinal stem cells against intestinal inflammation leading to epithelial regeneration in graft versus host disease patients who underwent bone marrow transplantation (56).

Another mechanism by which ILC regulates intestinal homeostasis is through their interaction with the commensal and/or pathogenic microbiota (48, 50, 57). Several protective mechanisms exist in the gut for the containment of commensal bacteria within intestinal sites including tight epithelial junctions, production of mucus and antimicrobial peptides, and immunological mechanisms that include ILC- and IgA-mediated immune exclusion pathways (58–63). ILC3 prevent commensal bacterial dysbiosis by IL-22-mediated induction of antimicrobial proteins (RegIIIβ, RegIIIγ, and β-defensins), element-sequestering proteins (S100A8, S100A9, and lipocalin-2) and mucins in epithelial cells leading to a strengthened intestinal epithelial barrier (49, 64–66). For example, depletion of ILC in mice led to selective peripheral dissemination of a commensal bacteria originating from host lymphoid tissues, namely *Alcaligenes* spp. and alcaligene-specific immune responses were found to be associated with Crohn’s disease and Hepatitis C virus-infected patients (63, 64). Further, ILC3 also are involved in the formation of gut-associated lymphoid tissues (GALT), including cryptopatches and isolated lymphoid tissues, which are important for protection against pathogens and act as niche areas of symbiosis for colonizing commensal microbiota (63). In turn, microbial products and signals were also found to be necessary for epigenetic modifications of ILC contributing to their diversity, plasticity, and maintenance of intestinal homeostasis (48, 57). This was evidenced by a study conducted by Manuzak et al. (67), describing the beneficial effects of probiotic therapy in healthy rhesus macaques by toll-like receptor (TLR) mediated downregulation of intestinal inflammatory markers and elevated ILC3 and T-follicular helper cells in colon.

Innate lymphoid cell can also act as a first line of defense at mucosal portals of entry due to their rapid production of cytokines following initial exposure to pathogens and recruitment of other innate and adaptive cells to sites of infection. ILC1 produce IFN-γ and TNF-α, both of which are important in the control of infections by intracellular pathogens such as *Toxoplasma gondii* (41) and *Listeria monocytogenes* (68). Furthermore, mice deficient in ILC3 were susceptible to intestinal pathogens including *Helicobacter* spp. and *Clostridium difficile* (69, 70). In helminthic infections, IL-25-mediated activation of ILC2 promotes a TH2 response which is important for an effective elimination of parasites (71). IL-17 is essential for the control of *Candida albicans* infection suggesting the importance of ILC3 in protection against oropharyngeal candidiasis in mice (72).

Given their critical roles in maintaining mucosal homeostasis, altered frequency or function of ILC during chronic disease could contribute to exacerbated intestinal inflammation. Indeed, intestinal ILC1 are elevated in IBD (73, 74), and production of IFN-γ by IL-15-activated ILC1 may play a major role in the pathogenesis of celiac and Crohn’s disease (25, 75). ILC2 along with NKT cells can also promote IL-13-mediated inflammation in an oxazolone-induced model of colitis (76). Interestingly, IL-23 responsive ILC3 can play a pathogenic role in intestinal inflammation through the production of IL-17A and IFN-γ and are also increased in patients with IBD (25, 28, 73, 74, 77–79). Given the significant protective roles ILC mediate in the GI tract, it may be important to take into account various interactions with intestinal epithelium and microbiota in achieving a balance of positive and negative ILC-related functions.

## Loss of ILC in Pathogenic Lentivirus Infections

One of the hallmarks of HIV and pathogenic SIV infection is early loss of gut integrity followed by massive and rapid translocation of microbial products from the lumen of the intestine into the lamina propria, blood, lymph nodes, and liver (80–83). Indeed circulating lipopolysaccharide (LPS), sCD14, and other microbial products are now well-established biomarkers for microbial translocation and immune stimulants associated with inflammation and chronic immune activation. Because ILC, particularly ILC3, play major roles in maintaining gut integrity, tissue modeling, and repair (53, 84–86), these cells are likely critical players in the pathophysiology of HIV/SIV disease. Initial work in SIV-infected rhesus macaques by our group and others showed that ILC3 are generally restricted to mucosal tissue, express high levels of RORγt, and produce IL-17 and IL-22 much like their human counterparts, but they are depleted or otherwise dysfunctional in infection (15, 16, 87). Specifically, we showed that even 1 week following SIV infection there was up to a threefold reduction in ILC3 in colon and fourfold to ninefold reduction in jejunum and ileum (19) and that this loss was maintained during chronic infection. Surprisingly, SIV viral loads did not correlate with the loss of ILC3 (19), nor were ILC3 infected *in vivo* (15).

Functionally, ILC3 from SIV-infected animals took on a more cytotoxic phenotype and produced greater quantities of TNF-α, IFN-γ, and MIP-1β, but reduced levels of IL-17 (14). This cytokine profile suggests lentivirus infection may drive ILC3 plasticity toward ILC1, as has been previously described for mice (10). Similarly, a study by Xu et al. (16) clarified the kinetic changes in IL-17-producing ILC3 from intestinal epithelium by showing a reduction during acute pathogenic SIV infection (7–14 days postinfection) is followed by an increase in the total numbers of ILC (14–21 days postinfection) and eventually a gradual decline of ILC3 with disease progression after 28 days postinfection (16). Klatt et al., (87) also noted a significant depletion of all IL-17-producing lymphoid cells in rhesus macaques, but not in sooty mangabeys, where SIV replicates efficiently but does not cause significant mucosal barrier damage. This observation further underscores a potential role for ILC3 in maintaining gut homeostasis in HIV/SIV infections. Work in an HIV model of humanized mice by Zhang et al. (88) showed that persistent HIV-1 infection depleted ILC3 but effective antiretroviral therapy reversed this loss.

In human subjects, Kloverpris et al. (89) found that all three subgroups of ILC in blood were depleted during infection, but early administration of ART restored all ILC subsets. However, if ART was not administered within 5–14 days after infection, only ILC3 were partially restored while ILC1 and ILC2 remained depleted. Much like had been shown in SIV-infected macaque models (14), ILC3 loss did not occur in tonsil or other oral mucosal tissues (89). Surprisingly, they did not detect a reduction of ILC numbers in the gut, and a similar observation was made by Fernandes et al. (90). Although ILC levels in the gut during acute infection were not measured. The reason for this discrepancy between these studies and multiple macaque studies are not clear, but could be species specific. This could also be the reason for the contrasting observations made by Liyanage et al. (91), suggesting no restoration of NKp44^+^ cells in the rectum after ART. More recently, a study by Kramer et al. (92) showed that intestinal ILC distribution is significantly perturbed in patients even on effective antiretroviral therapy and that levels of colonic ILC3 were inversely correlated to markers of microbial translocation.

One of the proposed mechanisms leading to mucosal inflammation in HIV infection is the interaction of viral envelope gp120 with polarized epithelial cells directly disrupting epithelial tight junctions (93–95). A closer look at the effect of viral infection on epithelial cells showed that HIV-1 directly reduces transepithelial resistance, a measure of epithelial cell monolayer integrity by 30–60% without affecting its viability (93). Furthermore, functions of tight junction proteins, such as claudin 1, 2, 4, occludin, and ZO-1, were also disrupted and significantly increased inflammatory cytokines, such as TNF-α, IL-6, MCP-1, and IL-1β (93). The resulting increase in cytokine production following T cell infection may also cause intestinal barrier breakdown [(96), reviewed in Ref. (97)].

The effect of HIV-2 on the other hand is less obvious. A previous study correlated both HIV-1 and HIV-2 with microbial translocation. However, a more recent study by Fernandes et al. (98) suggests no disruption of the epithelial tight junction by HIV-2 despite active replication. How ILC-mediated mucosal maintenance may differ in less pathogenic infections such as HIV-2 remains unstudied. Collectively, these data indicate that in both HIV-infected humans and pathogenic SIV-infected rhesus macaque models, ILC3 loss in the gut occurs early, is at least partially irreversible, and is linked to mucosal dysregulation and translocation of microbial products.

## Mechanisms of ILC Loss in Pathogenic HIV/SIV Infections

**Figure 1:**
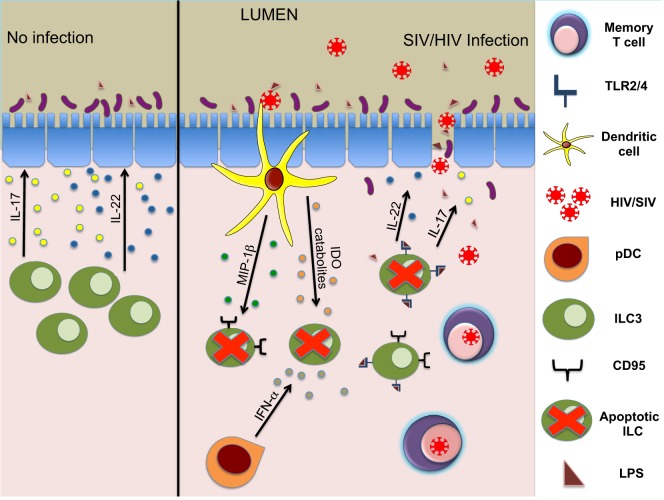
Mechanisms of ILC3 depletion during HIV/SIV infections. ILC3 modulate structure and homeostasis of gut epithelial cells *via* secretion of IL-17 and IL-22 (left panel). During the acute phase of lentivirus infection, early innate responders (DCs and other cells) secrete cytokines leading to apoptosis of ILC3. Subsequently, reduced IL-17 and IL-22 production leads to damage of gut epithelial barrier and an influx of microbial products, causing further inflammation (right panel).

While the loss of ILC during HIV/SIV infection is well established, multiple groups have pursued molecular and cellular mechanisms leading to this depletion. We had previously observed that the expression of IDO1, an enzyme upregulated during SIV infection [also observed in Ref. (99)] (Figure 1) correlates negatively with CD4^+^TH17 cells as well as ILC3 (15). In HIV, IDO has been implicated in immunosuppressive activity (100) and dysbiosis during disease progression (101). Although the source(s) of IDO1 are not totally clear, the ability of HIV-1 TAT to induce production of IDO catabolites by dendritic cells has been described previously (102). Interestingly, increased levels of IDO1 in the gut showed a negative correlation of CD4^+^ T cells and ILC3 but not with NK or CD8^+^ T cells (15). This suggested IDO1 expression could be a negative regulator of ILC3 but not other effector cells. Furthermore, we were able to confirm that IDO catabolites caused numerical and functional depletion of ILC3 through a similar mechanism described for TH17 cells (99). Increased apoptosis leading to massive loss in total numbers of ILC3 was observed; however, the loss was not due to direct infection as no detectable SIV RNA was present in these cells. This is not surprising, as ILC do not express receptors for SIV/HIV.

Further studies indicated that the loss of ILC3 in the mucosae during acute infection was due to increased apoptosis and RORγt suppression induced by inflammatory cytokines, such as TGF-β, IL-2, IL-12, and IL-15 (19). In pathogenic SIV infection, we also showed previously that plasmacytoid DC (pDC) accumulates in the gut mucosa producing large quantities of IFN-α (103) (Figure 1). HIV-1 infection in a humanized mouse model and *in vitro* treatment of splenic ILC3 with IFN-α or HIV-1 significantly upregulated CD95 expression on ILC3 leading to apoptosis dependent on pDCs (88). RNA-seq analysis of ILC in human subjects with acute HIV-1 infection showed that there was a downregulation of genes associated with viability (89), and gene array analysis (87) showed that mucosal IL-17^+^ cells highly expressed TNF-receptor superfamily 4 (TNFRSF4, OX40), a co-stimulatory molecule involved in maintenance of mucosal lymphocytes, in comparison to IL-17^−^ cells (104–106). Finally, ILC3 were shown to be depleted in lymphoid tissues mediated by TLRs in SIV-infected animals (107) (Figure 1). This study specifically showed that microbial translocation and resulting products like lipoteichoic acid or LPS *via* the TLR2/4 pathway can directly cause apoptosis in ILC3, further increasing HIV-induced disruption of GALT.

Altogether, these data indicated that a primary mechanism of ILC loss is likely apoptosis due to dysregulation of homeostatic elements on which ILC depend. One potential avenue that could be explored to restore ILC and gut integrity is IL-7-based therapies. Indeed in mice, IL-7 promoted IL-22 production during chronic LCMV infection (108); and in macaques, IL-7 therapy was shown to improve gut mucosal integrity in acute SIV-infected animals (109). Similarly, IL-7 immunotherapy in chronically infected HIV patients were associated with CD4^+^ T cell protective functions (108, 110, 111) and led to an overall reduced systemic inflammation (110). While these studies suggest that IL-7 plays a key role in repairing gut immunity, the precise connection to ILC is clearly understudied and needs further evaluation. Interestingly, it was also recently shown that SIV-ALVAC in combination with multiple adjuvants could induce an expansion of ILC3 (112). Whether or not this modality could be used therapeutically to restore ILC or could contribute to protective vaccine efficacy remains to be elucidated.

## Conclusion and Perspectives

Innate lymphoid cell fill a unique and plastic niche of primarily tissue-resident cells that provide innate sources of typical T cell and NK cell produced cytokines, and although they clearly have a role in innate defense and homeostasis, many unknowns remain. Not the least of which being a recent report indicating that individuals lacking ILC may experience no obvious pathology as long as an intact T and B cell compartment remains (113). Specifically, regarding lentivirus infections, infection itself is not the source of depletion, but rather indirect or direct apoptosis, and while some potential mechanisms have been described herein this list is unlikely exhaustive or complete. It is also important to note that in several HIV studies no ILC depletion is observed in the gut. Regardless, whether loss is a virus-mediated subversion or an off-target effect of massive inflammation is unclear, and although ILC3 seemingly mediate gut homeostasis, their exact roles, both kinetically and functionally, in the perturbation and subsequent microbial translocation following HIV and pathogenic SIV infections are not obvious. And given the tight reciprocal relationship between gut microflora and ILC3 in mice, it will be interesting to determine if ILC3 depletion also contributes to dysbiosis. Direct evidence for these phenomena will need to be confirmed by *in vivo* depletion strategies in macaques, should those reagents become available. Further, HIV/SIV clearly intersects with ILC3 but whether ILC2 and ILC1 also contribute against viral pathogenesis is less clear and will require further study. Nonetheless, despite a host of unknowns, the field as a whole can appreciate the novelty of these cell populations and conclude that manipulating ILC as early responders to infection could be an attractive target for multiple infectious as well as chronic conditions.

## Author Contributions

SS performed most of the writing and designed the figure. CM and DR contributed to writing of specific sections. RKR oversaw overall preparation of the manuscript, contributed to writing, and edited the final version.

## Conflict of Interest Statement

The authors declare that the research was conducted in the absence of any commercial or financial relationships that could be construed as a potential conflict of interest.
